# Chemical burns: pathophysiology and therapeutic protocols—do cervico-facial injuries pose specific challenges?

**DOI:** 10.25122/jml-2025-0082

**Published:** 2025-05

**Authors:** Rares-Adrian Giurgiu, Catalina-Stefania Dumitru, Andreea Grosu-Bularda, Eliza-Maria Bordeanu-Diaconescu, Adrian Frunza, Sabina Grama, Florin-Vlad Hodea, Tiberiu-Paul Neagu, Ioan Lascar, Cristian-Sorin Hariga

**Affiliations:** 1 Department of Plastic Surgery and Reconstructive Microsurgery, Carol Davila University of Medicine and Pharmacy, Bucharest, Romania; 2 Burn Centre, Emergency Clinical Hospital of Bucharest, Bucharest, Romania

**Keywords:** burns, chemical agents, therapeutic algorithm, dressings, surgical treatment

## Abstract

Chemical burns, though relatively rare, present significant diagnostic and therapeutic challenges due to their complex pathophysiology and the need for specialized care. A retrospective study was conducted, examining the characteristics, treatment, and outcomes of 33 patients with chemical burns admitted to our burn center for 8 years, representing 4.39% of all burn cases. Among them, 15 patients (45.45%) had chemical burns on the face and neck. The majority of these patients were men, with a relatively younger average age, and a significant proportion had work-related accidents. The burned surface area was variable, with many patients sustaining small total body surface area (TBSA), although some presented with extensive involvement. The prevalence of superficial partial-thickness burns was higher, but deep partial-thickness and full-thickness burns were also common. A large proportion of patients had favorable Abbreviated Burn Severity Index (ABSI) scores, indicating a high probability of survival. However, ocular involvement was a major complication. The study emphasizes the importance of timely intervention, including appropriate wound management strategies, specialized dressings, and skin substitutes. The findings also stress the need for a multidisciplinary approach, close monitoring, and adherence to safety protocols to optimize outcomes and minimize long-term complications in patients with chemical burns, particularly those of the face and neck region.

## INTRODUCTION

Burns are a significant global public health challenge, contributing to high morbidity and mortality rates. They often result in prolonged hospitalization and severe complications, whereas non-fatal cases frequently lead to chronic physical impairments such as disfigurement and disability, significantly impacting the quality of life of the burned survivors. These outcomes not only impose a substantial burden on healthcare systems but also have profound psychosocial impacts, including stigma and social exclusion [1-5].

Burn injuries are classified based on several factors, including depth, mechanism, and the percentage of body surface area affected. These classifications collectively determine the severity of the burn. The severity of burn injuries varies, and an increase in the affected total body surface area (TBSA) significantly impacts wound healing and increases patient mortality. [6,7]. Burns can be classified by depth into superficial, partial-thickness, or full-thickness. A superficial, first-degree burn affects only the epidermis. Superficial partial-thickness burns involve damage limited to the epidermis and the superficial dermis (Grade IIA), with most appendage structures intact, typically heal within 10–14 days with minimal risk of scarring. In contrast, deeper partial thickness (Grade IIB) burns extend into the deeper, reticular dermis, causing more extensive appendage damage. These burns take longer to spontaneously heal (more than three weeks) and carry a high risk of hypertrophic scarring. Full-thickness burns (third-degree burns) destroy all layers of the skin and require surgical treatment to ensure wound healing [6-8].

Regarding their mechanism, burn injuries result after exposure to high temperatures, electricity, friction, radiation, and various chemicals [6]. Among these types, chemical burns are a rare mechanism of injury (around 3.5%) but can cause severe damage depending on the nature of the chemical agent, its concentration, and the duration of exposure, posing significant morbidity and mortality risk [1,9]. Chemical burns are distinctive injuries that necessitate specialized management conducted in dedicated burn centers to ensure an adequate evaluation and treatment, depending on the specific causative agent [9-11].

Chemicals are widely produced for diverse purposes, including household, agricultural, industrial, and military applications. Effective management depends on categorizing these substances into broad classifications, although many chemicals may exhibit overlapping properties (Table 1). Chemical injuries frequently result from exposure to acids and alkalis, such as hydrofluoric acid, formic acid, anhydrous ammonia, cement, and phenol. Additional agents that can cause chemical burns include white phosphorus, elemental metals, nitrates, hydrocarbons, and tar. Currently, a wide variety of chemical substances are available, and each year, an impressive number of new agents with various purposes are introduced. However, the full extent of their potentially harmful effects on humans remains largely unknown [9,12-15].

**Table 1:** The classification of chemical agents based on their mechanism of action and their effects on biological systems [9,15]

Mechanism of action	Effects on tissues	Examples
**Reduction**	Binds free electrons in tissular proteins, provoking denaturation by reducing the amide link.	Hydrochloric acid, nitric acid, ferrous iron, sulphite compounds, alkyl mercuric compounds.
**Corrosive agents**	Denature tissue proteins upon contact, resulting in eschar formation and shallow ulceration.	Phenols, cresols, lye, sulfuric acid, hydrochloric acid, phosphorus, dichromate salts, and sodium metals.
**Oxidation**	Damages tissues by introducing oxygen, sulfur, or halogens into structural and functional proteins; produces toxic byproducts that further harm surrounding tissue.	Sodium hypochlorite, potassium permanganate, chromic acid, and peroxides.
**Protoplasmic poisons**	Disrupt tissue function by binding or inhibiting calcium and other essential ions; forms protein esters or chelates vital ions.	Acetic acid, hydrofluoric, formic, oxalic, and hydrazoic acid.
**Desiccants**	Cause tissue dehydration and exothermic reactions, leading to thermal injury and damage.	Sulfuric acid, hydrochloric acid (muriatic acid), calcium sulfate, and silica gel.
Vesicants	Cause ischemia and necrosis at the contact site, leading to cytokine release and blister formation.	Cantharides, mustard gas (sulfur and nitrogen), dimethyl sulfoxide (DMSO), and Lewisite (organo-arsenic compound).

Chemical burns are injuries that pose a significant diagnostic and therapeutic challenge, requiring specialized treatment promptly initiated by a well-trained multidisciplinary team. The patient’s prognosis and survival chances are heavily influenced by the timeliness and quality of care provided [1,6,9,10].

Among the various anatomical areas affected by burns, the head and neck regions present a unique and challenging situation. Facial burns result in severe functional impairments and significantly increase mortality risk when associated with airway injuries. For survivors, it profoundly impacts quality of life, leading to serious psychological distress and difficulties with social reintegration. A thorough understanding of the severity of head and neck burns is essential for effective acute management and preventing long-term complications [1,16-19].

This study aimed to characterize chemical burn injuries encountered in patients admitted to our burn center, to present the key principles of care, complications observed, and patient outcomes. In this study, we specifically analyzed patients with chemical burns in the head and neck regions, as these injuries pose distinct challenges. Also, an important objective was to detail the diagnostic and therapeutic strategies for managing these severe injuries.

## MATERIAL AND METHODS

We performed a retrospective study including patients who experienced chemical burns and were admitted to the Burn Unit of the Clinical Emergency Hospital Bucharest, between the 1^st^ of May 2016 and the 30^th^ of April 2024. All the patients included in the study were aged 18 and above and sustained chemical burns of at least grade IIA on a minimum of 1% body surface. The patients who did not present chemical burns or those with incomplete medical records were excluded from the study. The following data were collected: gender, age, the type of accident (domestic or work accident), the type of admission, length of hospitalization, comorbidities, the etiological agent of the chemical burn, the body surface area that was burned, the severity of the burn, involvement of the cephalic extremity, the type of surgical treatment, microbiological examination and outcome. The Abbreviated Burn Severity Index (ABSI) score was calculated to assess prognosis. Among this cohort of patients, the patients who presented with face and neck chemical burns were further selected, and a separate group of data was analyzed to determine the impact of face and neck chemical burns on the outcome. The collected data were analyzed using Microsoft Excel software.

## RESULTS

The study cohort included 33 patients with chemical burns, admitted to our burn center between the 1^st^ of May 2016 and the 30^th^ of April 2024, representing 4.39% of all burn patients. Among them, 15 patients (45.45%) had chemical burns on the face and neck. The characteristics of the cohort and sub-cohort of patients with chemical burns on the face and neck are depicted in Table 2.

**Table 2:** Characteristics of the patients in both study groups

Variables	Classification	Chemical burns patients (*n* = 33)		Sub cohort of face and neck chemical burns (*n* = 15)	
		Cases	Percentage	Cases	Percentage
Sex	Male	22	66.6%	11	43%
	Female	11	33.3%	4	27%
Accident type	Work	9	27%	5	33.3%
	Home	24	73%	10	66.6%
Comorbidities	Present	13	39%	4	27%
	Absent	20	61%	11	73%
Maximum burn depth	IIA	8	24%	6	40%
	IIB	14	43%	5	33%
	III	11	33%	4	27%
TBSA %	≤5%	20	61%	7	47%
	6% - 10%	5	15%	3	20%
	11% - 25%	4	12%	2	13%
	>25%	4	12%	3	20%
ABSI score	≤5	23	70%	12	80%
	6-9	8	24%	1	7%
	≥10	2	6%	2	13%

**Figure 1:**
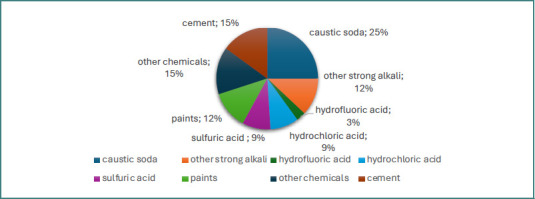
Etiological agents of chemical burns

Of all the patients with chemical burns, 27% (nine patients) sustained work-related accidents, and 18.18% (six patients) were admitted by transfer from other medical facilities lacking specialized burn care units. Most of the patients were men (66.6%), and the mean age was 45.6 years old, while the median age was 46. Of the 33 patients, 33.3% (11 patients) exhibited at least one full-thickness burn. Significant comorbidities were identified in 39% of the patients, most frequently cardiovascular (30.3% of patients), followed by renal (9%), metabolic (9%), pulmonary (6%), hepatic (6%), and neurological complications (6%). Regarding the burned surface area, 61% of the patients (20 patients) in the cohort sustained chemical burns covering less than 5% of the total body surface area, while 24% (eight patients) had chemical burns on more than 10% TBSA. The mean TBSA burned was 11% for the entire cohort, with a 15% TBSA for male patients and only 3% for female patients. In our study group, eight patients (24%) presented superficial partial-thickness burns, 14 patients (43%) had deep partial-thickness burns, and 11 patients (33%) had full-thickness chemical burns. The ABSI score was calculated, and it revealed that 70% of the patients had a probability of survival of 98% (ABSI score ≤5 points), while only 6% had a probability of survival below 40% (ABSI score ≥10 points). The average ABSI score was 5 points for both sexes (Table 2). The etiological agents of the chemical burns in the entire cohort of 33 patients are presented in Figure 1.

**Figure 2:**
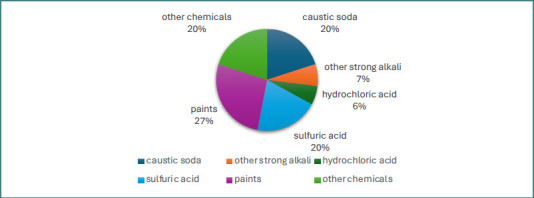
Etiologic agents of face and neck chemical burns

The group that sustained face and neck chemical burns had slightly different characteristics. Of these 15 patients, 33.3% (five patients) sustained work-related accidents. Among them, 73.3% (11 patients) were men, with an average age of 38.8 years old and a median age of 39 years. Significant comorbidities were identified in 26.6% of the patients, most frequently cardiovascular (13.3% of patients), followed by renal (6.6%), metabolic (6.6%), pulmonary (6.6%), and hepatic (6.6%). Regarding the burned surface area, the mean burned TBSA was 16%, with 47% of the patients (seven patients) having less than 5% of the total body surface area affected, while 20% (three patients) had chemical burns covering more than 25% of TBSA. Superficial partial-thickness chemical burns were more prevalent (40%), while 33% of patients presented deep-partial-thickness burns and 27% had full-thickness chemical burns of the face and neck. In this sub-cohort of patients, 80% of patients had an ABSI score ≤5 points, meaning a probability of survival over 98%, while 13% of patients had an ABSI score ≥10 points, which means a probability of survival of less than 40% (Table 2). Associated ocular burns were present in 53.3% of the patients, leading in one patient to lagophthalmos and aphakia. The etiological agents of the chemical burns in the sub-cohort of 15 patients with face and neck burns are presented in Figure 2.

According to the protocol in our clinic, microbiological screening was performed at admission to our burn unit. Of the 33 patients, 19 (57.6%) had positive cultures from the burn wounds upon admission, with seven patients identifying at least two bacterial species. The most frequently recorded pathogens at admission were coagulase-negative *Staphylococci*, found in 47.36% of these wounds, followed by *Staphylococcus aureus* and *Staphylococcus hominis*, each present in 15.8% of these cases. Infectious complications were recorded in three patients, leading to their exitus: one patient with tuberculosis and subsequent respiratory failure, and two patients with multiple organ dysfunction due to sepsis.

**Figure 3:**
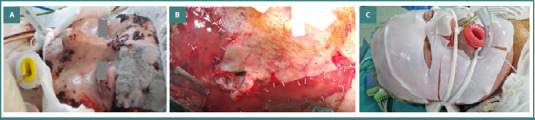
Advanced wound dressings in burn management. A, Aquacel Ag Burn Hydrofiber, a non-woven hydroentangled dressing comprised of hydrofiber (sodium carboxymethylcellulose) with nylon thread; B, Integra following a deep burn excision, the dermal matrix with the overlying silicone epidermal layer was applied; C, Epicite Hydro, a hydro-active dressing that creates a moist and supportive environment for wound healing used in a patient with chemical burns on the face.

**Figure 4:**
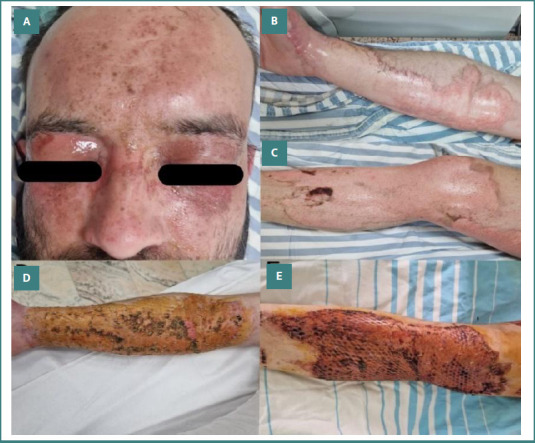
Sodium hydroxide (caustic soda) chemical burns and reconstruction. A, Acute full-thickness facial burns, including periocular involvement; B, upper limb; C, lower limb; D, Skin grafts in the upper limb; E, Skin grafts in the lower limb.

**Figure 5:**
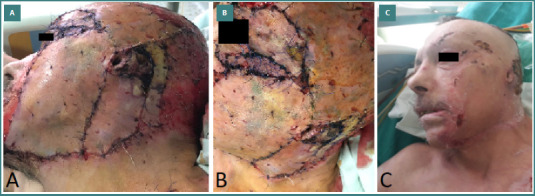
Reconstruction of facial chemical burns with split-thickness skin grafts. A, B Full-sheet split-thickness skin grafts used for definitive coverage after the excision of chemical burns on the face; C, Postoperative aspect on day 30 after skin grafting.

The therapeutic protocol in our burn unit includes specific measures in the emergency setting, followed by hospitalization in the ICU, where the patient benefits from a multidisciplinary treatment and rehabilitation program. The focus at admission is on stabilizing the patient, following the ABCD approach. Anamnesis should concentrate on identifying the incriminated chemical agent and the context of the burn injury, since specific local and systemic complications may occur. In all our patients, copious sterile water irrigation was started in the emergency department, and targeted therapeutic measures were initiated. In our case with hydrofluoric acid burn, calcium gluconate was administered intravenously, and by infiltrating the subcutaneous tissue with calcium gluconate solution to avoid dangerous arrhythmia, stabilization of the patient was successfully achieved. Debridement of the burn wound was performed, followed by wound dressing in most cases. In two cases, immediate surgical excision of eschars was performed. Clinical assessment of the burn depth was carried out on the following days, and further therapeutic decisions were made. The patients with partial-thickness chemical burns (24 patients, 72.7%) were treated conservatively, using a wide range of dressings. Full-thickness chemical burns benefited from surgical treatment in nine patients (27.3%), two of whom affected the head and neck area. The treatment consisted of surgical excision and definitive coverage using skin autografts and dermal substitute (Integra), followed by autografting in one case. Figures 3A-C, 4A-E, and 5A-C depict the above-mentioned therapeutic strategies.

The mortality rate within the study cohort was low, at only 9.09%. The average burn surface area in patients who died was 39.33% of TBSA. Additionally, all these patients had deep burns, including at least one full-thickness burn (grade III), and the ABSI score ranged from 4 to 11. All patients had work-related accidents and were transferred to our burn unit from other hospitals.

The length of stay (LOS) for the cohort of 33 patients varied significantly, with an average LOS of 17.36 days, and 66% of the patients leaving the hospital in the first 2 weeks. Among the patients with face and neck chemical burns, 80% of the patients were discharged in the first two weeks, and the average LOS was 18.8 days.

## DISCUSSION

Burn injuries are the fourth most common type of trauma worldwide, with high rates of incidence in low- and middle-income countries. Severe burns lead to shock and hypovolemia due to significant fluid loss, local and systemic inflammatory responses, hypermetabolism, and alterations in immune function. As a result, burn victims are at a higher risk for sepsis and other infections, single or multiple organ dysfunction, and an increase in morbidity and mortality [20-25].

Chemical burns constitute a severe and life-threatening condition that often results in significant esthetic and functional sequelae, or even death, representing 30% of the total burn-related fatalities. Managing such injuries requires meticulous attention and specialized efforts from the medical team and represents a burden on the healthcare system. Patients with chemical burns should be admitted to specialized burn units and undergo tailored treatment approaches, based on the causative agent, the extent of the burned surface, and the affected anatomical region [9,10,26,27].

This type of burn is relatively rare, accounting for only a few percent of all burn cases as reported in the literature [9,28]. This finding is consistent with our study, where chemical burn patients represented only 4.39% of the total number of patients admitted to our burn center. In addition, most cases involved male patients, with the most common circumstances being household-related incidents. However, work-related accidents also accounted for a significant percentage, representing 27% of the cases. The mean age of the patients was 45.6 years, with the majority being actively employed individuals. The average LOS in our study was 17.36 days, with two-thirds of the patients leaving the hospital in the first two weeks. The duration of hospitalization and the recovery period following such trauma pose significant challenges, incapacitating the burn victims socially and professionally and increasing the risk of complications in elderly patients. In addition to the substantial costs associated with hospitalization and specialized treatment, prolonged recovery periods often lead to extended work absences in younger patients. Moreover, patients may experience lasting functional and aesthetic sequelae, which can profoundly and permanently impact their quality of life and psychological well-being, even from a young age. The distribution by sex, age, and the environment in which the burns occurred in our study aligns with findings described in other studies [26,28-30]. This underscores the importance of protective measures in the workplace and highlights the need for proper education regarding the safe handling of chemicals in occupational settings and households.

The severity of chemical burns is rarely determined by the extent of the affected surface area; most cases involve a TBSA of less than 10% [29,30]. Instead, the mechanism of injury and the toxic properties of the chemical play a more important role. This is supported by our findings, where the mean total body surface area burned was 11%, with 61% of patients having a TBSA involvement of under 5%, and only 24% having burns exceeding 10% TBSA. Nevertheless, the majority of burns in our study were severe, with 76% of patients experiencing deep partial-thickness or full-thickness burns.

Another important feature of chemical burns is the frequent involvement of the head and neck, as documented in several medical studies and papers on this subject [10,27-31]. In our study, burns affecting the head and neck were observed in 45.45% of the patients, a significant proportion. Half of these patients also sustained ocular burns. Although most facial burns do not require surgical intervention, the involvement of such an esthetically and functionally critical area demands specialized care. Ocular involvement can lead to permanent vision impairments, including blindness [28,29,32]. Furthermore, burns in this anatomical area carry the additional risk of airway injuries, digestive tract damage in the case of ingestion, and systemic toxicity. [10,28,30,33,34]. Airway involvement can occur either directly, through the inhalation of the causative agent, or indirectly, due to severe edema that may compress the upper airways [35]. The toxicity of the chemical substance can be exacerbated if it comes into contact with the respiratory or gastrointestinal mucosa via aspiration or ingestion, leading to increased absorption and heightened systemic toxicity [33]. These findings underscore the critical need to raise public awareness about the importance of using proper protective equipment when handling chemicals at home and highlight the necessity for robust regulations and standardized safety protocols at the workplace.

Regarding the type of causative chemical agent, the main substance involved was caustic soda, incriminated in a quarter of all cases in both sexes, followed by cement, accounting for 15.15% of cases. For patients with burns on the face and neck, the situation was slightly different, with the primary substances involved being paint in 27% of cases, followed by caustic soda in 20% of cases, and sulfuric acid in another 20% of cases. Sodium hydroxide (caustic soda) is one of the most commonly involved substances in chemical burns, as described in clinical studies [28,29,31,34]. It is a strong alkaline substance found in many households, causing both accidental and self-inflicted burns. It is important to note that caustic soda-induced ocular burns are extremely severe since they rapidly penetrate the cornea, leading to opacity, scarring, or even perforation at this level [28]. Additionally, as a substance commonly found in households, caustic soda is frequently involved in severe chemical burns in children due to accidental ingestion [36,37]. Calcium oxide is the agent responsible for chemical burns from contact with cement. When calcium oxide reacts with water, it forms calcium hydroxide, and the hydroxyl ion induces skin damage, resulting in liquefaction necrosis [28]. The fact that paints were the most frequently involved substances in facial chemical burns raises concerns about the regulation of substances that are accessible to the general public without proper safety measures or adequate training.

At admission to the burn unit, 57.6% of patients had positive bacteriological cultures from the burn wounds, with seven patients identifying at least two bacterial species. *Staphylococcus* species were the most common, similar to other reports in the literature [38-41]. The prompt diagnosis and treatment of infections in burn patients is crucial, as there is a proportional relationship between the number of bacterial species present and the length of hospitalization for these patients [38,39]. In our study, infectious complications occurred in three patients, resulting in their deaths: one patient succumbed to tuberculosis and subsequent respiratory failure, while two others developed multiple organ dysfunction due to sepsis.

The mortality rate in the study cohort was relatively low (9.09%). All of them were victims of work-related accidents and were transferred from other healthcare facilities lacking specialized burn units. They sustained burns to the cephalic region, and the mean total body surface area affected by burns was 39.33%. Additionally, all these patients presented deep burns, including at least one full-thickness burn.

**Figure 6:**
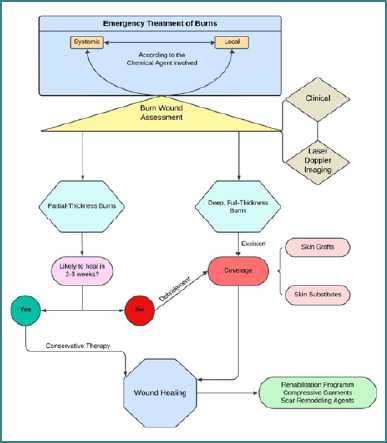
Therapeutic algorithm of chemical burns: particularities of cervico-facial burns

Based on current recommendations in the field, we have developed a comprehensive protocol specifically designed to treat chemical burns. This protocol provides a structured approach to managing such injuries effectively, ensuring optimal outcomes. The key steps are schematically illustrated in Figure 6, describing the treatment process and the underlying principles [1,9,42-46].

Partial-thickness burns usually benefit from conservative treatment using topical strategies to promote wound healing. Particularly, chemical burns in the head and neck region present unique challenges in non-surgical management due to their complex anatomical and functional implications. These injuries may result from assaults, where motives include social or personal disputes, leading to severe outcomes such as vision loss and contractures [47,48].

The management of such burns requires adherence to established trauma protocols, emphasizing airway protection, hemodynamic stability, and infection prevention [49].

Non-surgical treatment of head and neck chemical burns involves an approach that prioritizes immediate decontamination, wound care, and supportive therapies. Initial management typically includes thorough irrigation with water to remove the chemical agent, although care must be taken with certain chemicals like alkalis, where water can exacerbate the injury through exothermic reactions [9,50,51].

Acute management also aims to minimize long-term complications, such as hypertrophic scarring and contractures, which can lead to functional impairments like microstomia and ectropion [17].

In cases of hydrofluoric acid burns, immediate washing with water is crucial, and topical applications of calcium and magnesium compounds are recommended to neutralize fluoride ions and prevent deep tissue damage [52,53].

Regular dressing changes and topical agents, such as silver sulfadiazine, are common practices to promote healing and prevent infection [54].

More recent therapies, such as biodegradable matrices and skin substitutes, have shown promise in improving wound healing outcomes and may be considered in resource-limited settings where surgical options are not readily available [55].

Textile dressings have shown potential utility in the treatment of head and neck chemical burns, primarily due to their ability to deliver drugs locally and maintain a moist wound environment, which is crucial for healing. For dressings used in head and neck chemical burns, a knitted backing layer and a hydrogel layer with silver sulfadiazine may optimize swelling and porosity, ensuring efficient drug delivery and wound coverage [56,57].

As the knowledge of wound healing grows, advancements in treatment options and technologies for managing second-degree burns continue to progress. Polymeric hydrogels represent a category of burn wound dressings that offer multiple benefits, including tissue adherence, absorption of wound exudate, environmental protection, and transparency, allowing ongoing wound assessment. Additionally, some hydrogels are designed for easy removal during dressing changes [58].

Hydrogels in textile dressings, as described in the studies, offer a promising approach for burn treatment. These hydrogels, often loaded with antibiotics like silver sulfadiazine, provide a controlled release of medication directly to the wound site, enhancing the healing process by preventing infection and promoting tissue regeneration [56,59,60].

Additionally, incorporating essential oils and other antimicrobial agents into hydrogels further enhances their antibacterial properties, making them effective against common pathogens such as *S. aureus* and *E. coli* [59].

The design of these dressings, which includes layers that manage exudate while maintaining a moist interface, is particularly beneficial for burn wounds, as it facilitates optimal healing conditions. Moreover, the adaptability of these dressings to various body parts, including the face, suggests their applicability in treating head and neck burns [61].

The advancements in medical textiles, including the use of nanofibers, have also contributed to improved wound healing by providing mechanical support, facilitating gas exchange, and maintaining temperature homeostasis [62,63]. Overall, integrating advanced textile technologies in burn care represents a significant step forward in managing chemical burns in sensitive areas like the head and neck.

In high exudative lesions, after accurate debridement, negative pressure wound therapy may drain excess fluids from the burn, minimize the progression of the wound, and reduce inflammation. The association of silver nanoparticle dressings brings additional benefits, such as the antimicrobial effect, and provides a favorable environment for wound healing [64,65].

For deep and full-thickness chemical burns, adequate treatment involves early excision and grafting [66].

Skin grafting on the face requires special considerations due to its unique anatomy, aesthetic significance, and functional importance. The facial skin has a highly vascularized structure, which generally ensures better graft acceptance and healing. Achieving optimal functional and aesthetic outcomes is crucial, as the face is central to a person’s identity and social interactions. Unmeshed split-thickness grafts are preferred for large burn areas, while full-thickness grafts may be used for smaller, critical regions such as the eyelids, nose, or lips to minimize scarring and ensure a better color and texture match. Donor sites must be carefully selected to provide skin that closely resembles the facial area in terms of pigmentation and thickness. A meticulous surgical technique is essential to provide proper alignment of facial landmarks and avoid distortion of critical features. More complex defects may require reconstruction using loco-regional flaps or free microvascular transfers. Postoperative care, including scar management and rehabilitation, plays a crucial role in optimizing both functional and aesthetic outcomes [9,18, 46, 67-70].

Serious burns of the head and neck often lead to predictable facial deformities, including neck contraction limiting movement, chin contraction causing lower lip eversion and oral competence issues, microstomia, upper lip shortening, nasal tip retraction, medial canthal webs, eyelid ectropion, and texture or pigmentation abnormalities. Additionally, hypertrophic or keloid scars may develop, sometimes raising cosmetic concerns without functional impairment. These patients often require multiple staged reconstructive surgical interventions to achieve optimal therapeutic outcomes. Therapeutic adherence and careful follow-up are essential [18].

## CONCLUSION

Chemical burns of the head and neck are complex injuries that require a specialized, multidisciplinary approach to management. The causative agent and mechanism of injury have a greater impact on the severity of these burns than the total body surface area affected. Early intervention, including prompt irrigation, debridement, and targeted therapies, is essential for minimizing morbidity and mortality. Advanced wound care technologies, such as hydrogels, skin substitutes, and textile-based dressings, have demonstrated significant benefits, particularly in the non-surgical management of these injuries. Early excision and grafting remain the cornerstone of treatment for deeper burns and should be complemented by innovative reconstructive techniques to optimize functional and aesthetic outcomes. Preventive strategies, including stringent safety protocols and public education on chemical handling, are crucial for reducing the incidence of these injuries. A comprehensive therapeutic protocol, supported by long-term follow-up and rehabilitation, is fundamental to addressing both functional and psychosocial challenges and enhancing the overall quality of life for affected patients.

## References

[1] Jeschke MG, van Baar ME, Choudhry MA, Chung KK, Gibran NS, Logsetty S (2020). Burn injury. Nat Rev Dis Primers.

[2] Yin B, He Y, Zhang Z, Cheng X, Bao W, Li S (2024). Global burden of burns and its association with socio-economic development status, 1990-2019. Burns.

[3] Badoiu SC, Enescu DM, Tatar R, Miricescu D, Stanescu-Spinu II, Greabu M (2024). Adipokines-A Cohort Prospective Study in Children with Severe Burns. Int J Mol Sci.

[4] Parry I, Bell J (2024). Associations between burn care services and impairment at discharge after burn injury: Analysis of the Global Burn Registry. Burns.

[5] Bordeanu-Diaconescu EM, Grosu-Bularda A, Frunza A, Grama S, Andrei MC, Neagu TP (2024). Diagnostic and Prognostic Value of Thrombocytopenia in Severe Burn Injuries. Diagnostics (Basel).

[6] Żwierełło W, Piorun K, Skórka-Majewicz M, Maruszewska A, Antoniewski J, Gutowska I (2023). Burns: Classification, Pathophysiology, and Treatment: A Review. Int J Mol Sci.

[7] Warby R, Maani CV (2024). Burn Classification. [Updated 2023 Sep 26]. StatPearls [Internet].

[8] Schaefer TJ, Szymanski KD (2024). Burn Evaluation and Management. [Updated 2023 Aug 8]. StatPearls [Internet].

[9] Williams FN, Jong OL, Herndon DN (2018). Chemical Burns. Total Burn Care.

[10] American Burn Association Guidelines for Burn Patient Referral. [Internet].

[11] Eftekhari H, Sadeghi M, Mobayen M, Esmailzadeh M, Feizkhah A, Lahiji MS (2023). Epidemiology of chemical burns: An 11-year retrospective study of 126 patients at a referral burn centre in the north of Iran. Int Wound J.

[12] Touzopoulos P, Zarogoulidis P, Mitrakas A, Karanikas M, Milothridis P, Matthaios D (2011). Occupational chemical burns: a 2-year experience in the emergency department. J Multidiscip Healthc.

[13] Pruitt VM (2006). Work-related burns. Clin Occup Environ Med.

[14] Edlich RF, Farinholt HM, Winters KL, Britt LD, Long WB, Werner CL, Gubler KD (2005). Modern concepts of treatment and prevention of chemical injuries. J Long Term Eff Med Implants.

[15] VanHoy TB, Metheny H, Patel BC (2024). Chemical Burns. [Updated 2023 Jul 17]. StatPearls [Internet].

[16] Charles WN, Collins D, Mandalia S, Matwala K, Dutt A, Tatlock J (2022). Impact of inhalation injury on outcomes in critically ill burns patients: 12-year experience at a regional burns centre. Burns.

[17] Shams Ortiz A, Chan RK, Dion GR (2020). Skin burns of the head and neck. Oper Tech Otolaryngol Head Neck Surg.

[18] Burd A (2010). Burns: treatment and outcomes. Semin Plast Surg.

[19] Ryan CM, Lee A, Stoddard FJ, Li NC, Schneider JC, Shapiro GD (2018). ; Multi-Center Benchmarking Study Group. The Effect of Facial Burns on Long-Term Outcomes in Young Adults: A 5-Year Study. J Burn Care Res.

[20] Peck MD (2011). Epidemiology of burns throughout the world. Part I: distribution and risk factors. Burns.

[21] Badoiu SC, Enescu DM, Tatar R, Stanescu-Spinu II, Miricescu D, Greabu M (2024). Serum Plasminogen Activator Inhibitor-1, α 1-Acid Glycoprotein, C-Reactive Protein, and Platelet Factor 4 Levels-Promising Molecules That Can Complete the “Puzzle” of the Biochemical Milieu in Severe Burns: Preliminary Results of a Cohort Prospective Study. J Clin Med.

[22] Bordeanu-Diaconescu EM, Grosu-Bularda A, Frunza A, Grama S, Andrei MC, Neagu TP (2024). Venous Thromboembolism in Burn Patients: A 5-Year Retrospective Study. Medicina (Kaunas).

[23] Burgess M, Valdera F, Varon D, Kankuri E, Nuutila K (2022). The Immune and Regenerative Response to Burn Injury. Cells.

[24] Asim M, Amin F, El-Menyar A (2020). Multiple organ dysfunction syndrome: Contemporary insights on the clinicopathological spectrum. Qatar Med J.

[25] Bordeanu-Diaconescu EM, Grosu-Bularda A, Frunza A, Grama S, Andrei MC, Dumitru CS, Costache RA, Marinescu BM, Lascar I (2024). Comprehensive review and update on multiple organ dysfunction syndrome in burn patients: pathophysiology, risk factors, and management strategies. Rom J Mil Med.

[26] Walsh K, Hughes I, Dheansa B (2022). Management of chemical burns. Br J Hosp Med (Lond).

[27] Jurkiewicz MJ (1990). Plastic Surgery: Principles and Practice. Mosby.

[28] Palao R, Monge I, Ruiz M, Barret JP (2010). Chemical burns: Pathophysiology and treatment. Burns.

[29] Abbasi H, Dehghani A, Mohammadi AA, Ghadimi T, Keshavarzi A (2021). The Epidemiology of Chemical Burns Among the Patients Referred to Burn Centers in Shiraz, Southern Iran, 2008-2018. Bull Emerg Trauma.

[30] Ye C, Wang X, Zhang Y, Ni L, Jiang R, Liu L (2016). Ten-year epidemiology of chemical burns in western Zhejiang Province, China. Burns.

[31] Li W, Wu X, Gao C (2013). Ten-year epidemiological study of chemical burns in Jinshan, Shanghai, PR China. Burns.

[32] Grant WM (1950). Chemical burns of the eye. J Am Med Assoc.

[33] Kang S, Kufta K, Sollecito TP, Panchal N (2018). A treatment algorithm for the management of intraoral burns: A narrative review. Burns.

[34] Cartotto RC, Peters WJ, Neligan PC, Douglas LG, Beeston J (1996). Chemical burns. Can J Surg.

[35] Tanizaki S (2015). Assessing inhalation injury in the emergency room. Open Access Emerg Med.

[36] Nguyen ATM, Chamberlain K, Holland AJA (2021). Paediatric chemical burns: a clinical review. Eur J Pediatr.

[37] D’Cruz R, Pang TCY, Harvey JG, Holland AJA (2015). Chemical burns in children: Aetiology and prevention. Burns.

[38] El Hamzaoui N, Barguigua A, Larouz S, Maouloua M (2020). Epidemiology of burn wound bacterial infections at a Meknes hospital, Morocco. New Microbes and New Infections.

[39] Roy S, Mukherjee P, Kundu S, Majumder D, Raychaudhuri V, Choudhury L (2024). Microbial infections in burn patients. ACC.

[40] Weinand C (2024). Associated bacterial and fungal infections in burn wounds: Common factors, distribution in etiology, age groups, bacterial and fungal strands–Evaluation of a single burn center experience of 20 years. Burns Open.

[41] Pangli H, Papp A (2019). The relation between positive screening results and MRSA infections in burn patients. Burns.

[42] Nielson CB, Duethman NC, Howard JM, Moncure M, Wood JG (2017). Burns: Pathophysiology of Systemic Complications and Current Management. J Burn Care Res.

[43] Monstrey SM, Hoeksema H, Baker RD, Jeng J, Spence RS, Wilson D (2011). Burn wound healing time assessed by laser Doppler imaging. Part 2 validation of a dedicated colour code for image interpretation. Burns.

[44] Singer AJ, Boyce ST (2017). Burn Wound Healing and Tissue Engineering. J Burn Care Res.

[45] Papini R (2004). Management of burn injuries of various depths. BMJ.

[46] Jeschke MG, Shahrokhi S, Finnerty CC, Branski LK, Dibildox M, ABA Organization & Delivery of Burn Care Committee (2018). Wound Coverage Technologies in Burn Care: Established Techniques. J Burn Care Res.

[47] Saeidinia A, Keihanian F, Lashkari AP, Lahiji HG, Mobayyen M, Heidarzade A (2017). Partial-thickness burn wounds healing by topical treatment: A randomized controlled comparison between silver sulfadiazine and centiderm. Medicine (Baltimore).

[48] Tahir C, Ibrahim BM, Terna-Yawe EH (2012). Chemical burns from assault: a review of seven cases seen in a Nigerian tertiary institution. Ann Burns Fire Disasters.

[49] Jennes S, Hanchart B, Keersebilck E, Rose T, Soete O, François PM (2016). Management of burn wounds of the head and neck region. B-ENT.

[50] Claes KEY, Vyncke T, De Wolf E, Hoeksema H, Verbelen J, Monstrey S (2020). Enzymatic debridement as an effective treatment for combined flame and chemical burns caused by e-cigarettes. Am J Emerg Med.

[51] Friedstat J, Brown DA, Levi B (2017). Chemical, Electrical, and Radiation Injuries. Clin Plast Surg.

[52] McKee D, Thoma A, Bailey K, Fish J (2014). A review of hydrofluoric acid burn management. Plast Surg (Oakv).

[53] Zhang Y, Wang X, Liu Y, Jiang X, Ye C, Ni L (2017). Management of a Rare Case With Severe Hydrofluoric Acid Burns: Important Roles of Neutralizers and Continuous Renal Replacement Therapy. Int J Low Extrem Wounds.

[54] Galo A, Farid M, Almasharqah R (2022). The conservative management of self-inflicted chemical burns: Case report and literature review. Scars Burn Heal.

[55] Varon DE, Carlsson AH, Cooper LE, Chapa J, Valdera FA, Christy S (2023). Evaluation of Topical Off-The-Shelf Therapies to Improve Prolonged Field Care of Burn-Injured Service Members. Mil Med.

[56] Cuturicu LL, Macarel VC, Rusu RA, Lacatusu C, Danila A, Statescu L (2023). A textile device for the therapy of patients with burn wounds by the use of a drug delivery from a hydrogel to dermis. J Eng Fibers Fabr.

[57] Visileanu E, Ene A, Mihai C, Vladu A (2023). Textile structures for the treatment of burn wounds–characterization of elastic and antibacterial properties. Industria Textila.

[58] Cook KA, Martinez-Lozano E, Sheridan R, Rodriguez EK, Nazarian A, Grinstaff MW (2022). Hydrogels for the management of second-degree burns: currently available options and future promise. Burns Trauma.

[59] Georgiana D, Vasile A, Tigau A, Popescu R, Constantinescu L, Chirila L (2022). Hydrogels-based textile materials for treatment of first-degree burn injuries. ICAMS 2022-9^th^ International Conference on Advanced Materials and Systems.

[60] Surowiecka A, Strużyna J, Winiarska A, Korzeniowski T (2022). Hydrogels in Burn Wound Management-A Review. Gels.

[61] National Center for Biotechnology Information PubChem Patent Summary for WO-2008001100-A2. Dressings for treating burns.

[62] Chen S, Liu B, Carlson MA, Gombart AF, Reilly DA, Xie J (2017). Recent advances in electrospun nanofibers for wound healing. Nanomedicine (Lond).

[63] Liu X, Xu H, Zhang M, Yu DG (2021). Electrospun Medicated Nanofibers for Wound Healing: Review. Membranes (Basel).

[64] Dumville JC, Munson C, Christie J (2014). Negative pressure wound therapy for partial-thickness burns. Cochrane Database Syst Rev.

[65] Abu-Baker A, Țigăran AE, Peligrad T, Ion DE, Gheoca-Mutu DE, Avino A (2024). Exploring an Innovative Approach: Integrating Negative-Pressure Wound Therapy with Silver Nanoparticle Dressings in Skin Graft Procedures. J Pers Med.

[66] Hoogewerf CJ, Hop MJ, Nieuwenhuis MK, Middelkoop E, Vlies CH, Van Baar ME (2012). Early excision and grafting for burns. Cochrane Database Syst Rev.

[67] Samuel J, Gharde P, Surya D, Durge S, Gopalan V (2024). A Comparative Review of Meshed Versus Unmeshed Grafts in Split-Thickness Skin Grafting: Clinical Implications and Outcomes. Cureus.

[68] Donelan MB, Bojovic B, Herndon DN (2018). Reconstruction of the head and neck after burns. Total Burn Care.

[69] Hofer SO, Payne CE (2010). Functional and Aesthetic Outcome Enhancement of Head and Neck Reconstruction through Secondary Procedures. Semin Plast Surg.

[70] Jabir S, Frew Q, El-Muttardi N, Dziewulski P (2014). A systematic review of the applications of free tissue transfer in burns. Burns.

